# Occupational Therapy Interventions in Patients with Frontotemporal Dementia: A Systematic Review

**DOI:** 10.3390/medsci11040071

**Published:** 2023-11-06

**Authors:** Pinelopi Vlotinou, Anna Tsiakiri, Georgia Detsaridou, Alexandrina Nikova, Dimitrios Tsiptsios, Konstantinos Vadikolias, Nikolaos Aggelousis

**Affiliations:** 1 Department of Occupational Therapy, University of West Attica, 12243 Athens, Greece; pvlotinou@uniwa.gr; 2 Neurology Department, Democritus University of Thrace, 68100 Alexandroupolis, Greece; georgia.detsaridou@gmail.com (G.D.); dtsiptsi@med.duth.gr (D.T.); kvadikol@med.duth.gr (K.V.); 3 Department of Neurosurgery, Democristus University of Thrace, 68100 Alexandroupolis, Greece; nikovaalex@gmail.com; 4 Department of Physical Education and Sport Science, Democritus University of Thrace, 69100 Komotini, Greece; nagelous@phyed.duth.gr

**Keywords:** occupational therapy, interventions, frontotemporal dementia, quality of life

## Abstract

Frontotemporal dementia (FTD) is a neurodegenerative disorder characterized by progressive impairments in behavior, executive function, and language, primarily affecting individuals under the age of 65. This disorder is associated with expressive and receptive anomia, word comprehension deficits, and behavioral symptoms such as apathy, loss of empathy, and disinhibition, all of which closely correlate with functional impairment in daily activities. Despite substantial efforts, research on occupational therapy (OT) interventions has yet to demonstrate clear benefits in managing the disease. The aim of this study is to investigate OT interventions and assess their efficacy, with a specific focus on individuals suffering from FTD. We systematically conducted searches on two databases, namely Medline and Science Direct, spanning a ten-year period from 2003 to 2023, in accordance with the PRISMA guidelines. Eleven studies met the inclusion criteria. OT interventions targeted both patients and caregivers and yielded significant positive improvements in their lives. A key focus of these interventions was to teach acceptable alternatives to the behaviors exhibited by FTD patients, as these behaviors are strongly influenced by the disease itself. OT contributes positively to enhancing the quality of life of FTD patients and alleviating the caregiving burden experienced by those providing long-term care to these patients.

## 1. Introduction

FTD represents a complex spectrum of neurodegenerative disorders characterized by a range of symptoms, including progressive impairments in behavior, executive function, and speech. Notably, FTD is more prevalent among individuals under the age of 65 [1,2,3]. Within the landscape of FTD, three primary clinical variants have been meticulously delineated.

Behavioral Variant FTD (bvFTD). This variant often manifests with early and prominent behavioral and executive deficits. Individuals afflicted with bvFTD experience personality changes, delinquency, and profound apathy [4]. Their behavior can be marked by social inappropriateness, characterized by a lack of manners, rudeness, and impulsive movements [5]. Shockingly, some patients exhibit severe antisocial behaviors, including theft, sexual harassment, and public urination. Executive function deficits are common in this variant, although visuospatial skills remain relatively preserved during the initial stages. Notably, many patients with bvFTD lack insight into their behavioral changes and may not recognize the magnitude of these transformations. Binge eating, increased consumption of sweets or alcohol, and subsequent weight gain are additional facets of the behavioral-variant FTD [1,2,6,7].

Non-Fluent Variant of Primary Progressive Aphasia (nfvPPA). This variant is characterized by progressive speech deficits, encompassing difficulties with grammar and word output. Intriguingly, despite substantial impairments in complex language and executive task processing, individuals with nfvPPA often retain the capacity to comprehend simple sentences with a semantically similar content [1,2,8].

Semantic Variant Primary Progressive Aphasia (svPPA). In svPPA, a syndrome defined by semantic aphasia and associative agnosia emerges. During the initial stages of the disease, patients may present with grammatically correct speech and maintain a generally pleasant conversational demeanor [1,8].

As FTD inexorably progresses, it is conceivable that these initially distinct variants begin to overlap, converging into a clinical presentation characterized by common end-stage symptoms. These end-stage manifestations encompass profound difficulties in eating, mobility, and swallowing [1,8].

OT stands as a cornerstone in the comprehensive care of individuals grappling with dementia, including FTD. The benefits of OT programs in this context are multifaceted, encompassing substantial enhancements in activities of daily living and instrumental activities of daily living when compared to conventional care. Beyond these practical improvements, OT interventions have been shown to reduce behavioral and psychological symptoms, significantly elevating the overall quality of life for FTD patients [9]. Importantly, OT extends its reach to encompass the invaluable collaboration with families, offering guidance, education, and active participation in the care process. This holistic approach aids families in redefining their relationships with their loved ones affected by FTD. Simultaneously, it fosters a parallel pathway to support caregivers, enriching both the lives of those suffering from FTD and the caregivers themselves. By expertly managing caregiver burden through targeted OT interventions, caregivers are empowered to navigate the challenges posed by FTD with greater resilience, minimizing the personal loss and fatigue they may experience [9,10].

However, despite the promise and potential of non-pharmaceutical interventions such as OT in managing FTD, it is essential to acknowledge that references to these approaches remain limited and under-evaluated in the current scientific literature. Thus, the primary objective of this study is to comprehensively explore and assess occupational therapy approaches and programs specifically tailored to individuals facing the complex challenges of FTD. Through this investigation, we aim to shed light on the potential of OT interventions in enhancing the lives of those afflicted by FTD and their caregivers.

## 2. Materials and Methods

The Preferred Reporting Items for Systematic Reviews and Meta-analyses (PRISMA statement) was used to guide this study [11], conducting the last search on 21 October 2023 (Registration number: CRD42023474151).

### 2.1. Search Strategy

Literature research of three databases (Medline, Science Direct, Scopus) was conducted by two independent researchers (PV and AT), tracing all relevant studies published between 2003 and 2023, using the terms “Frontotemporal dementia” AND “Occupational Therapy” as keywords. 

### 2.2. Selection Criteria

We identified the search terms by abstract’s title. After duplicate removal, manuscripts were evaluated based on abstract and text. The criteria that determined the suitability of the research articles were the following:

- Original full-text articles;
- Articles published in English language;
- Peer-reviewed studies;
- Case studies;
- Study protocols were excluded.

### 2.3. Data Recorded

An excel form was created to record information concerning the manuscript’s authors, the year of publication, the title and the research type, the methodology that was followed in each case, and finally, the results of every research.

### 2.4. Data Analysis

The Newcastle–Ottawa quality assessment scale (NOS) for cohort studies (https://www.ohri.ca, assessed on 4 April 2023) was performed for the included studies. Based on the NOS star rating system, we rated seven items classified into three categories: selection of study groups, comparability of the groups, and ascertainment of outcome of interest. Each study was awarded a maximum of one star for each item within the selection and outcome categories, while a maximum of two stars were given for the comparability category. The maximum for each study is nine stars (9), with studies having more than six stars being identified as representing a low risk of bias. Scores of 6–9, 3–5, and 0–2 points represented high, moderate, and low quality, respectively. The data were only descriptively analyzed, while no statistical analysis or meta-analysis was performed.

Forest plots were employed to visualize the effect measure of individual studies, which provide the mean and standard deviation (SD) of the data in experimental groups (after intervention). Each study has its own standardized mean difference, which is calculated by taking the difference in the group means and dividing by the standard deviation of the groups. If the diamond shape does not touch the line of no effect, the difference found between the two groups was statistically significant. The plot in these figures suggest that there are different effect sizes in different types of interventions.

## 3. Results

### 3.1. Database Searches

**Figure 1:**
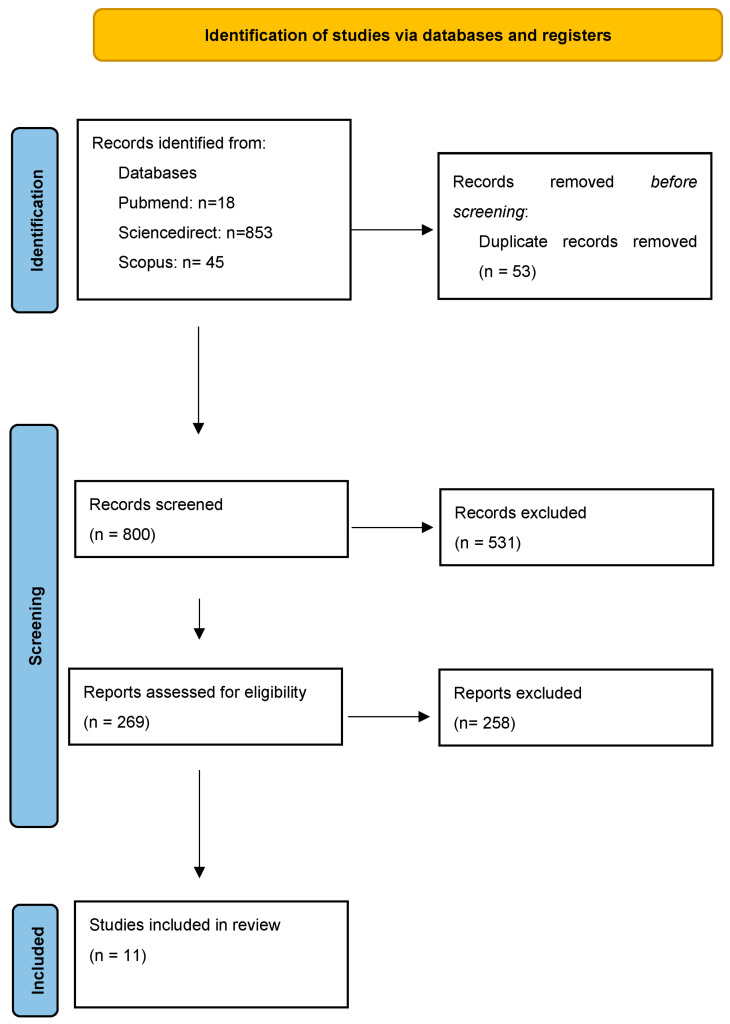
Study flow diagram (PRISMA flowchart).

Overall, 916 records were retrieved from the database search. We excluded duplicates and irrelevant studies and a total of 269 articles were selected. After screening the full texts of the articles, 11 studies fulfilled the eligible criteria for inclusion (Figure 1). Six studies were categorized as high-quality, four studies were categorized as moderate-quality, and one as low-quality (Table 1).

### 3.2. Results on Behavioral Management in Occupational Therapy for FTD

The effectiveness of OT in addressing the unique challenges of FTD has been explored through two distinct perspectives: classical occupational therapy practice with a client-centered approach to group occupational therapy and individualized work facilitation. In a study conducted by O’Connor et al. [23], the focus was on establishing effective behavioral management strategies within the framework of OT intervention to yield positive outcomes (Table 2).

In this study, a group of 21 FTD patients became the focal point of research. The objective was to devise a structured approach to behavioral management within the scope of OT. This approach is particularly crucial for FTD patients because they may exhibit impulsive, socially inappropriate, and challenging behaviors. The assessment process employed in this study involved a multifaceted evaluation. First, a comprehensive functional assessment was conducted to understand the specific behaviors and difficulties exhibited by each patient. Second, observations were made within the patients’ home environments, allowing for a more holistic understanding of their daily challenges and triggers. Following these assessments, the occupational therapists collaborated closely with the patients and their caregivers to create personalized behavioral support plans. These plans were not one-size-fits-all; instead, they were tailored to the individual needs and circumstances of each patient and their respective caregivers.

A key aspect of these plans was their focus on neuropsychological rehabilitation behaviors, which encompass cognitive and emotional aspects of functioning. Furthermore, the interventions placed a significant emphasis on environmental modifications, making changes in the home environment to better suit the patients’ needs and minimize stressors. The approach was grounded in positive reinforcement, which means that it aimed to encourage and reward desirable behaviors while simultaneously addressing and minimizing challenging or unwanted behaviors. This comprehensive approach seeks to enhance the quality of life for both patients and their caregivers by effectively managing the behavioral aspects of FTD.

In the study of Langhamer et al. [22], an exploratory design was employed to assess the impact of an 8-week combined intervention involving physical activity, music therapy, and daily walking on individuals with severe dementia and frontal lobe symptoms in institutional care. The primary outcome measure, evaluated using the Brøset Violence Checklist (BVC), revealed significant improvements (*p* = 0.03) in anxiety, restlessness, irritability, and aggression among the participants. This study demonstrates the feasibility of implementing individualized music therapy alongside increased physical activity, indicating its efficacy in reducing behavioral symptoms associated with severe dementia.

In the same line, the study of Yokota [21] investigated the impact of relocating patients with frontotemporal dementia (FTD) from a traditional psychiatric ward to a group home setting with a focus on a more homelike and patient-centered approach. Before relocation, patients exhibited behavioral symptoms including disinhibition, aggression, and agitation. The study assessed cognitive function, the behavioral and psychological symptoms of dementia (BPSD), quality of life (QOL), and psychotropic drug use in FTD patients over a 6-month period. The results indicated that the group-home care approach significantly improved BPSD, as evidenced by reduced NPI and CMAI scores. Additionally, several aspects of QOL showed improvements, and the use of psychotropic drugs decreased. While further research with larger samples is needed, these findings suggest the value of a patient-centered and homelike care environment in managing behavioral disturbances in FTD patients, potentially enhancing their well-being.

The study of Ferrero-Arias et al. [19] found a high prevalence of apathy in the dementia patients, estimated at around 70%, which is consistent with observations in other studies. Apathy was strongly correlated with the severity of cognitive decline and the overall severity of dementia, indicating that its prevalence increases with the progression of dementia. In contrast, there was no significant relationship between depression and cognitive loss or overall severity, highlighting the distinction between apathetic and depressive disorders in dementia. The study revealed that structured occupational therapy interventions, such as music therapy, improved apathy in dementia patients, especially those with mild-to-moderate apathy. However, the effect was less pronounced in patients with severe apathy. Patients assigned to the initial intervention group showed a slight advantage in maintaining the positive effects beyond the intervention period. The therapists found that music therapy was particularly effective in actively engaging patients in the intervention. The study suggests that the intervention may have lasting benefits and emphasizes the need for further research to determine the optimal duration, frequency, and specific components of such interventions for different groups of dementia patients.

**Figure 2:**
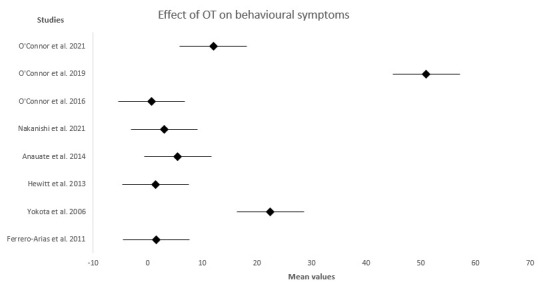
Forest plot showing the effect of Occupational Therapy interventions on behavioral symptoms. O’ Connor et al., 2021 [12]; O’Connor et al., 2019 [13]; O’Connor et al., 2016 [15]; Nakanishi et al., 2021 [14]; Anauate et al., 2014 [16]; Hewitt et al., 2013 [18]; Yokota et al., 2006 [21]; Ferrero-Arias et al., 2011 [19].

In summary, these studies highlight the crucial role of occupational therapy in addressing the complex behavioral challenges associated with frontotemporal dementia. In Figure 2, there is a representation of the therapeutic interventions used in the analysis and how they positively affected the reduction of neuropsychiatric and behavioral symptoms. By tailoring interventions to individual needs and emphasizing positive strategies, this approach can lead to improved outcomes and a better quality of life for both patients and their caregiver.

### 3.3. Results on the Intersection of Behavior Management and Caregiver Support

In a parallel vein, O’Connor et al. [15] conducted a study involving individuals afflicted by FTD, with a sample size of 2 (*n* = 2), focusing on the dual aspects of behavior management and caregiver support. Specifically, OTs implemented the Tailored Activities Program (TAP), a bespoke approach designed to mitigate socially unacceptable behaviors in individuals with FTD. Data collection encompassed cognitive assessments, evaluations of functional disability, as well as an assessment of caregiver confidence and vigilance. These assessments occurred both before and after the implementation of TAP.

Regarding the impact of TAP on patients, the findings revealed an increased level of participation in activities, which in turn positively correlated with improvements in functionality and behavior management. Additionally, the occupational therapy program had a favorable effect on caregivers’ confidence levels. Furthermore, participants demonstrated a high level of engagement in the prescribed activities, as evidenced by reduced distress among caregivers regarding challenging behaviors and an enhanced sense of caregiver vigilance. O’Connor et al. revisited the TAP intervention in 2019, expanding their study to include 20 dyads (*n* = 40) comprising individuals suffering from FTD and their respective caregivers. Qualitative analysis yielded five prominent themes: “perceived benefits for caregivers”, “readiness of caregivers to embrace change”, “strategies employed by caregivers to engage with the Tailored Activity Program”, “obstacles encountered in the adoption and implementation of the Tailored Activity Program”, and “participation of individuals with dementia”. The quantitative results indicated a general decrease in patients’ behavioral symptoms and sustained levels of functional performance post TAP intervention [13].

Grinberg et al. [17] presented the FTD Day Program at Baycrest, which focuses on enhancing the quality of life for individuals with FTD. The program provides strategies and opportunities to increase self-worth and accomplishment among its members while fostering a caring, friendly, and trusting environment. It operates from Monday through Friday, offering secure door-to-door transportation within a designated catchment area. The program features tailored individual and small-group activities that stimulate members, provide support, and restore a sense of independence. It also offers essential respite for families and provides education and support for both members’ families and caregivers. The interdisciplinary team utilizes behavior management techniques and a supportive, enabling approach to address the unique needs of FTD members and their families.

One of the critical outcomes of the program is the establishment of strong collaborative relationships between the FTD Day Program and the Memory Disorders Clinic, creating a more comprehensive continuum of care. Staff members have ongoing contact with nurse clinicians in the Memory Disorders Clinic, enhancing the services and support available to members and their families. The day center offers crucial support to caregivers by providing respite for families. It also provides education and support to the families and caregivers of the members. The center has a highly trained interdisciplinary team that uses behavior management techniques and a supportive approach to address the needs of the FTD members and their families. They offer education on behavioral modification techniques, specific features of FTD, home safety, ways to stimulate loved ones at home, and stress management. Social workers play a key role in engaging the entire family system, helping family members reconnect, and understanding the complexity of the illness. The center encourages families to seek additional support and normalization in their lives. Particularly for younger members, this support is essential, with social workers maintaining a positive outlook and motivating staff to enhance the lives of members and their families through various activities and approaches.

The impact of the FTD Day Program extends beyond Baycrest, as it aims to disseminate best practices. The program has shared its experiences and expertise through publications and presentations, both in the United States and Canada, to promote universal best practices in working with similar day programs internationally. The development of the program continues as an iterative process, with ongoing planning and refinement to meet the evolving needs of FTD patients and their families.

A more recent study [23], while primarily focused on the benefits of a Positive Behavior Support (PBS) intervention for individuals with FTD, indirectly highlights the positive impact on caregivers. The study showed that the intervention was associated with improvements in the severity of apathetic and disinhibited behaviors, which can be challenging for caregivers to manage. The reduction in caregiver distress regarding apathy suggests that caregivers also experienced benefits from the PBS approach, as it helps them develop better management and coping abilities.

The case study [14] emphasizes the use of routinizing therapy to address stereotyped behavior in individuals with FTD, which can be beneficial for caregivers. The reduction in Behavioral and Psychological Symptoms of Dementia (BPSD) implies a decreased caregiving burden. The study shows how caregiver support is closely linked to the management of challenging behaviors in FTD patients. 

Finally, researchers [18] focused on the effects of group gardening activities for individuals with Young-Onset Dementia (YOD), which indirectly highlights benefits for caregivers. Carers reported improved self-identity, suggesting that they experienced reduced caregiver stress and improved well-being. Additionally, structured group activities provided caregivers with valuable respite.

These studies collectively indicate that caregiver support in the context of occupational therapy interventions for FTD patients is crucial. Interventions not only aim to benefit the patients themselves, but also have positive outcomes for caregivers. Reducing caregiver distress, lightening caregiving duties, and enhancing caregiver well-being are essential aspects of these interventions. However, it is important to acknowledge that caregiver support remains a central component of the holistic care approach for individuals with FTD.

### 3.4. Activities of Daily Life–Quality of Life

Nakanishi and Yamaga [14] conducted a comprehensive study to evaluate the impact of implementing a structured program for daily activities on the overall quality of life. Their research centered on a case study involving a woman afflicted with FTD residing in a group home. The patient exhibited a range of distressing symptoms, including agitation, apathy, disinhibition, irritability, and stereotyped behaviors. These symptoms not only diminished the patient’s quality of life, but also placed an increased burden on her caregivers. The OT program aimed to establish and execute a structured daily routine that encompassed various activities and instrumental tasks within the home environment. The introduction of a well-defined routine for household chores notably alleviated the behavioral and psychological symptoms associated with FTD, thereby reducing the long-term care burden on the patient’s caregivers. The primary focus of the study was to enhance the quality of life for the patient. Consequently, their findings demonstrated that implementing a structured routine for Activities of Daily Living could effectively benefit both individuals suffering from dementia and their caregivers. This intervention resulted in an improvement in the psychological well-being and overall quality of life for the individuals with dementia, while simultaneously significantly reducing the caregiving burden.

A case report [20] focused on a 60-year-old man who experienced a 2–3-year history of functional decline and behavioral changes, which initially led to a misdiagnosis of a late-onset psychiatric disorder. The patient underwent a full workup for neurodegenerative diseases during his second inpatient admission, ultimately resulting in a diagnosis of probable PSP-F (Progressive Supranuclear Palsy with a frontal lobe cognitive or behavioral presentation). The report detailed the patient’s neurological rehabilitation plan, comparing recommendations made before and after the correct diagnosis. Following the diagnosis of the neurodegenerative disorder, significant changes were observed in the neurological rehabilitation plan, particularly in physical therapy (PT) and occupational therapy (OT), despite no alterations in the patient’s clinical presentation. This emphasized the critical role of an accurate and early diagnosis in tailoring rehabilitation strategies. Furthermore, the report highlighted the patient’s longitudinal improvement in an OT session, underlining the importance of rehabilitation in the lives of individuals with PSP-F. The study concluded that increasing the recognition of PSP variants among healthcare providers is vital to ensure early and appropriate diagnoses. This, in turn, allows patients to receive optimal neurological rehabilitation plans, maintain their quality of life, and extend their functioning periods.

Another study [18] indicated a gradual increase in positive indicators of well-being over the course of the structured gardening program for individuals with young-onset dementia. It was observed that participation in the gardening program helped maintain well-being levels, even as cognitive function declined over the 12-month study period. The most common themes mentioned by carers were improved self-identity, purposeful activity, and mood. Participants felt a sense of belonging to the group and derived a sense of achievement and purpose from their gardening activities. Additionally, improved self-identity and group belonging appeared to positively impact participants’ mood. The study suggested that therapeutic gardening has the potential to enhance the well-being, cognition, and mood of individuals with young-onset dementia. 

Anauate et al. [16] focused on how individuals with FTD exhibit distinct profiles in their artistic tasks. Their drawings displayed simpler compositions, were poorly and rapidly executed in copy and collage tasks, and they exhibited an altered use of colors and details in comparison to the model. These deficits in artistic performance could be linked to impairments in visuospatial perception, organization, and thinking operations. The study used an ecological assessment method that provides a more functional evaluation of cognitive function. Tasks such as copying and collage revealed specific cognitive deficits in FTD patients, particularly in areas related to visuoconstructional and executive functions. Qualitative evaluation was also emphasized in the study. It was observed that FTD patients, particularly those with behavioral variant FTD (bvFTD), had impairments related to impulsivity and difficulties in task duration, planning, and organization. Semantic variant PPA (PPA) patients, on the other hand, tended to produce more line-based artwork with less recognition of the picture content, while non-fluent PPA patients displayed better performance. This exploratory study suggests that artistic tasks can serve as alternative tools for assessing cognitive dysfunction in FTD patients, particularly when traditional neuropsychological testing may be challenging due to behavioral disturbances. Further research with larger samples and a focus on early-stage FTD may provide deeper insights into the cognitive profiles of different FTD subtypes. Additionally, understanding the underlying processes in art production can contribute to optimizing rehabilitation efforts, including art therapy, for individuals with FTD.

**Figure 3:**
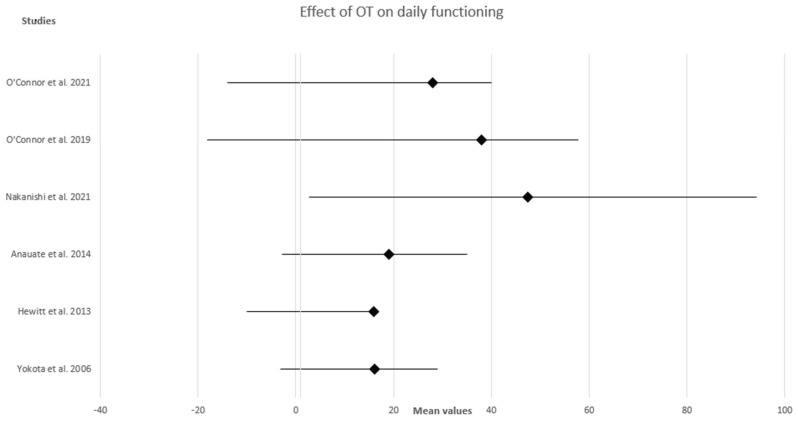
Forest plot showing the effect of Occupational Therapy interventions on activities of daily living and cognitive functioning. O’ Connor et al., 2021 [23]; O’ Connor et al., 2019 [13]; Nakanishi et al., 2021 [14]; Anauate et al., 2014 [16]; Hewitt et al., 2013 [18]; Yokota et al., 2006 [21].

Figure 3 shows the overall effect of the therapeutic strategies included in our analysis on improving activities of daily living and cognitive functioning.

### 3.5. Results οn Recommendations Raised from This Review

Intervention Approaches: The studies highlight the importance of tailored intervention approaches. PBS and TAP have shown promise in addressing challenging behaviors in individuals with FTD. Non-drug therapies that focus on IADL are also recommended [13,14,23].

Impact on Caregivers: Caregivers play a critical role in the well-being of individuals with FTD. The studies suggest that intervention approaches not only benefit the individuals, but also have a positive impact on caregivers. Caregivers experience improved skills and confidence, resulting in reduced time spent on caregiving duties. Group activities, such as gardening, can provide respite for caregivers and contribute to the well-being of patients [13,18,23].

Subtype Considerations: FTD is a heterogeneous condition and the studies emphasize the need to tailor interventions according to FTD subtypes. Behavioral goals may differ based on whether the individual has behavioral variant FTD (bvFTD) or semantic dementia (SD). Additionally, different levels of impairment are observed in amyotrophic lateral sclerosis (ALS), FTD-ALS, and bvFTD, suggesting that interventions should be customized accordingly [15,23].

Physical and Social Environment: Creating a supportive physical and social environment is crucial for individuals with FTD. Homelike environments have been shown to improve BPSD, enhance quality of life, and reduce the use of psychotropic drugs. Group activities in non-hospitalized settings can improve self-identity, mood, and provide a sense of purpose for patients [21,22].

Need for Early Diagnosis: Early and appropriate diagnosis is emphasized as it allows for the tailoring of neurological rehabilitation plans. Early intervention can have a significant impact on the effectiveness of treatment and the overall well-being of individuals with FTD [19,20].

Behavior Changes and Cognitive Functions: Understanding the remaining emotional and cognitive functions of individuals with FTD is essential. The studies recommend considering these factors when designing interventions. Additionally, interventions, such as art therapy, need to account for cognitive deficits, such as visuospatial perception and thinking operations [14,16].

## 4. Discussion

Despite the significance of FTD due to its profound impact on patients’ lives and the substantial burden it places on caregivers, there remains a critical gap in our understanding of the effectiveness of OT interventions aimed at enhancing the quality of life for FTD patients. Some studies have honed in on tailored activity programs capable of reducing or managing the typical behavioral and psychological manifestations of dementia, ultimately improving the quality of life, reducing caregiving demands, and enhancing the well-being of family caregivers. Others advocate for behavioral treatments within OT programs. For instance, Oliveira et al. [24] underscored the effectiveness of the TAP in reducing neuropsychiatric symptoms in both dementia patients and their caregivers. They reported significant reductions in hallucinations, anxiety, aggression, agitation, sleep disorders, and caregiver burden, suggesting TAP as a potent non-pharmacological strategy for addressing behavioral and neuropsychiatric symptoms in dementia.

The impact of a combined intervention explored involving physical activity, music therapy, and daily walking on individuals with severe dementia and frontal lobe symptoms [22]. This intervention showed significant improvements in anxiety, restlessness, irritability, and aggression among participants. It suggests the feasibility and efficacy of implementing individualized music therapy alongside increased physical activity to reduce behavioral symptoms associated with severe dementia. In a similar vein, researchers investigated the benefits of relocating FTD patients from traditional psychiatric wards to a more patient-centered, homelike group home setting [21]. This approach significantly improved behavioral and psychological symptoms of dementia (BPSD), quality of life, and reduced psychotropic drug use. The findings support the value of a patient-centered care environment in managing behavioral disturbances in FTD patients, potentially enhancing their well-being. Lastly, Ferrero-Arias et al.’s [19] study highlighted the high prevalence of apathy in dementia patients, with a strong correlation between apathy, cognitive decline, and dementia severity. Structured occupational therapy interventions, including music therapy, were effective in improving apathy, particularly in patients with mild-to-moderate apathy. This study emphasizes the need for further research to determine the optimal duration, frequency, and specific components of such interventions for different groups of dementia patients.

OT can play a pivotal role in significantly improving the lives of individuals grappling with FTD. The primary aim of OT interventions is to contribute positively to the rehabilitation of patients’ behavior. Moreover, OT interventions encompass key goals such as enhancing patients’ overall quality of life, slowing the progression of the disease, and lessening the long-term caregiving burden. Behavioral management strategies within OT can effectively target disruptive behaviors, stereotypic behaviors, and even inappropriate speech. Other studies highlighted the value of reintroducing old habits, hobbies, or games as part of “routinizing therapy”, which is a systematic approach embedded in OT programs [25,26]. While cognitive training is proposed as an alternative behavioral treatment, it is not typically considered the primary choice for FTD treatment [27,28]. 

As FTD progresses, maintaining participation in activities of daily living becomes increasingly challenging. To achieve these goals, occupational therapists recommend the application of TAP, which has demonstrated efficacy in reducing behavioral symptoms associated with FTD. Individuals treated with TAP are better able to engage in everyday activities, reaping multiple benefits.

In line with this, OT is strongly advocated as one of the most specialized disciplines for implementing ergonomic adjustments in dementia care. When motor difficulties surface, occupational therapists play a pivotal role by recommending ergonomic changes to the environment, enhancing patient safety and independence within their living spaces.

Beyond these mentioned approaches, several other interventions aim to slow the progression of FTD and improve quality of life [16,18,20]. Physical activity, for instance, has emerged as a multifaceted treatment strategy. By maintaining higher levels of physical activity, mobility issues, stability concerns, and balance difficulties can be mitigated. However, it is worth noting that there is limited information available on physical activity interventions from the perspective of OT [29]. 

An organized living environment, where every object has a designated place, can help individuals maintain control over their surroundings and daily routines. Home safety evaluations, coupled with fall prevention measures, prove especially valuable for FTD patients with apraxia or fine motor manipulation impairments [4]. This structured routine fosters familiarity with activities, significantly improves psychological well-being, and elevates overall quality of life. Engagement in daily activities is often employed in the care of dementia patients, with reminiscing and calming techniques through activity forming integral components of OT approaches in managing behavioral and psychological symptoms.

In conclusion, the application of occupational therapy protocols can greatly enhance the quality of life for individuals affected by FTD. Consequently, there is a pressing need to organize and implement a wider range of protocols across a larger sample of the population affected by this debilitating condition.

### Strengths and Limitations

The strengths of this review include its adherence to the PRISMA guidelines, which ensures a systematic and transparent approach to conducting the study. The comprehensive search strategy, involving three databases and an examination of references, enhances the likelihood of capturing relevant studies. The well-defined selection criteria and use of an Excel form for data recording contribute to the rigor and clarity of the review. Furthermore, the inclusion of a quality assessment using the Newcastle–Ottawa scale adds credibility by evaluating the risk of bias in the included studies. The review’s descriptive data analysis and discussion of key findings provide valuable insights into the role of occupational therapy in managing behavioral challenges associated with frontotemporal dementia.

However, there are some limitations to this review. It focuses on a specific aspect of frontotemporal dementia and a specific intervention, which limits its scope and may not provide a comprehensive overview of all relevant interventions and outcomes. The small sample size of included studies raises concerns about the generalizability of the findings. Finally, a broader exploration of diverse occupational therapy interventions could have enriched the review’s perspective.

## 5. Conclusions

In conclusion, this systematic review, guided by the PRISMA statement and robust methodology, sheds light on the role of occupational therapy in addressing the intricate behavioral challenges associated with frontotemporal dementia (FTD). The strengths of this review lie in its comprehensive search strategy, transparent selection criteria, and meticulous quality assessment, all contributing to a well-structured and informative analysis. It underscores the significance of tailored interventions and behavioral management within occupational therapy in improving the quality of life for individuals with FTD and alleviating the caregiving burden.

These results hold relevance for clinicians, caregivers, and policymakers involved in the care of individuals with FTD. Occupational therapy, with its focus on personalized interventions and holistic approaches, emerges as a pivotal discipline in enhancing the well-being of both patients and their caregivers. By emphasizing positive strategies, cognitive rehabilitation, and environmental modifications, occupational therapy offers a promising avenue for managing the behavioral and psychological symptoms of FTD.

### Future Challenges

In the realm of FTD care and research, there are several pressing challenges that necessitate attention in the coming years. One fundamental challenge is the standardization of intervention approaches. While studies have shown promise in addressing challenging behaviors in individuals with FTD through methods like PBS and TAP, there is a need for a greater consensus on evidence-based and standardized approaches. Such standardization would ensure more consistent and effective care for FTD patients. Additionally, the heterogeneity of FTD, with subtypes like bvFTD and SD, presents a challenge in terms of tailoring interventions. The distinct characteristics and symptoms associated with each subtype require tailored approaches to address the unique challenges they pose.

Caregiver support is another crucial area where future efforts are needed. Caregivers play a pivotal role in the well-being of individuals with FTD, but they often face significant stress and burden. Providing them with better support, resources, and training is essential to enhance their ability to manage the care of FTD patients effectively. Early diagnosis and screening are paramount in FTD, yet they remain challenges. Improving diagnostic tools and increasing awareness among healthcare providers can lead to early detection and intervention, which significantly impacts patient outcomes. As FTD is a progressive condition, long-term care models need to be developed and refined to provide sustainable and high-quality care as the disease advances. Ensuring that patients continue to experience a high quality of life throughout the course of their illness is of utmost importance.

A comprehensive understanding of the cognitive deficits associated with FTD subtypes is also required. Future research should delve deeper into these deficits and develop targeted strategies to address them effectively. Exploring the potential benefits of combining non-pharmacological interventions, such as behavioral support and activity programs, with pharmacological treatments or other therapies is another avenue for future investigation. These combined approaches may offer more comprehensive care for FTD patients. The impact of the physical and social environment on FTD patients is an area that requires further exploration. Studies have indicated that homelike environments and group activities can influence behavior, quality of life, and overall well-being. However, a more nuanced understanding of these impacts is needed.

Measuring and reporting behavioral changes is a challenge due to the heterogeneity of FTD and the need for consistent assessment tools. Developing standardized measures and ensuring that both objective and subjective data align consistently is essential for the accurate reporting of treatment outcomes. Lastly, multidisciplinary collaboration is vital in addressing the challenges associated with FTD. Neurologists, psychiatrists, occupational therapists, and other healthcare professionals must work together to provide holistic care for individuals with FTD. Collaboration can lead to more comprehensive and effective care plans.

In conclusion, addressing these future challenges requires a coordinated effort from various stakeholders, including researchers, healthcare providers, caregivers, and policymakers. By tackling these challenges, we can improve the quality of care and support available to individuals affected by FTD and enhance their overall well-being and quality of life.

## Figures and Tables

**Table 1:** Quality assessment of included studies by Newcastle–Ottawa scale.

	Selection				Comparability	Outcome		
Study	Exposed Cohort	Non-Exposed Cohort	Ascertainment of Exposure	Outcome of Interest		Assessment of Outcome	Length of Follow-Up	Adequacy of Follow-Up
O’Connor et al. [12]	-	-	√	√	√	√	-	√
O’Connor et al. [13]	√	√	√	√	√√	√	-	√
Nakanishi K. and Yamaga T. [14]	-	-	√	√	-	√	-	√
O’Connor et al. [15]	-	-	√	√	-	√	-	√
Anauate et al. [16]	-	√	√	√	√	√	-	-
Grinberg et al. [17]	-	-	-	√	-	√	-	-
Hewitt et al. [18]	√	-	√	√	√√	√	√	√
Ferrero-Arias et al. [19]	√	√	√	√	√√	√	-	√
Reisch et al. [20]	-	-	√	√	√√	√	√	√
Yokota et al. [21]	-	-	√	√	√√	√	√	√
Langhammer et al. [22]	-	-	√	√	√√	√	-	√

**Table 2:** Characteristics of included studies.

Author (1^st^)	Title	Participants	Methodology	Results
O’Connor [12]	Positive behaviour support in FTD: A pilot study	N = 21	Assessments included a combination of qualitative and quantitative measures to assess program acceptance, daily living, and behavioral symptoms.	Target behaviors typically served a “sensory” function, which suggests that these behaviors are pleasurable (self-reinforcing) to individuals. Often people avoid a situation or a task they do not want to do. Interestingly, people with FTD are socially withdrawn and avoid bustling places.
O’Connor [13]	The tailored activity program (TAP) to address behavioral disturbances in FTD: a feasibility and pilot study	N = 40	The Tailored Activity Program was performed. Qualitative analyses assessed the feasibility and acceptability of the program on five domains: “perceived benefits by the caregiver”, “readiness of the caregiver for change”, “strategies used by the caregiver to provide for a person with dementia”, “barriers to uptake/implementation of the Adapted Activity Program”, and “Participation of people with dementia”.	Quantitative results showed an overall reduction in behavioral symptoms and maintenance of functional performance in the person with dementia.
Nakanishi [14]	Effect of Instrumental Activities of Daily Living habituation due to routinising therapy in patients with FTD	N = 1	Examined whether Major Activities of Daily Living improve with routine therapy for a patient with FTD. The patient showed symptoms of agitation, apathy, disengagement, irritability, and stereotyped behavior.	It is suggested that the routine in the Major Activities of Daily Living of the patient with FTD reduced the burden of long-term care and improved the patient’s quality of life.
O’Connor [15]	Enhancing caregivers’ understanding of dementia and tailoring activities in FTD: two case studies	N = 2	Described the intervention process and results of the Tailored Activities Program in two individuals diagnosed with FTD.	The first subject, after the implemnmetation of TAP intervention, could consistently participate in more activities, with scores indicating improvements in caregiver behavior, function, and confidence. The second subject was engaged well in intended activities, with scores reflecting reduced caregiver distress about challenging behaviors and improved caregiver vigilance after TAP program.
Anauate [16]	Performance of patients with frontotemporal lobar degeneration on artistic tasks	N = 4	FTD patients were assessed using the Lowenstein Occupational Therapy Cognitive Assessment (LOTCA) and by a three-stage artistic protocol including visual observation, copying, and collage, based on a Sisley painting.	FTLD patients presented impairment in the visuospatial and executive skills required to perform artistic tasks. We demonstrated that the application of the instrument as a complimentary method for assessing cognitive skills in this group of patients is possible.
Grinberg [17]	Multidisciplinary Design and Implementation of a Day Program Specialized for the Frontotemporal Dementias	N = 6	The study involved the development of an innovative day program for individuals with Frontotemporal Dementia (FTD). The primary objective was to enhance the quality of life for program members, their families, and caregivers.	The program features tailored individual and small-group activities that stimulate members, provide support, and restore a sense of independence.
Hewitt [18]	The Efficacy of Nonpharmacological Treatment forDementia-related Apathy	N = 145	A controlled, cross-over, randomized, simple-blind, multicenter clinical trial in which both intervention and control periods lasted for 4 weeks. Occupational therapy sessions occurred on weekdays and lasted 50 min each, following a 3-day rotation scheme, including music therapy, art therapy, psychomotor activity, and mime, to prevent boredom, until a total of 20 sessions had been completed.	The study revealed that structured occupational therapy interventions, such as music therapy, improved apathy in dementia patients, especially those with mild-to-moderate apathy. However, the effect was less pronounced in patients with severe apathy.
Ferrero-Arias [19]	Does a Structured Gardening Programme Improve Well-Being in Young-Onset Dementia? A Preliminary Study	N = 12	Over a year, the participants attended 46 weekly sessions, engaging in gardening tasks tailored to their abilities. The sessions were led by a team of healthcare professionals, including an occupational therapist and horticultural therapist.	The study revealed that an appropriate gardening program, tailored to individual cognitive and physical abilities, had a positive impact on participants’ lives, even when there was no significant change in their clinical presentation.
Reisch [20]	Increasing awareness of rare PSP-F, where rehabilitation is the primary treatment: A case report	N = 1	The patient underwent a full workup for neurodegenerative diseases during his second inpatient admission, ultimately resulting in a diagnosis of probable PSP-F (Progressive Supranuclear Palsy with a frontal lobe cognitive or behavioral presentation). The report detailed the patient’s neurological rehabilitation plan, comparing recommendations made before and after the correct diagnosis.	Following the diagnosis of the neurodegenerative disorder, significant changes were observed in the neurological rehabilitation plan, particularly in physical therapy (PT) and occupational therapy (OT), despite no alterations in the patient’s clinical presentation. This emphasized the critical role of an accurate and early diagnosis in tailoring rehabilitation strategies. Furthermore, the report highlighted the patient’s longitudinal improvement in an OT session, underlining the importance of rehabilitation in the lives of individuals with PSP-F.
Yokota [21]	Effects of Group-Home Care on Behavioral Symptoms, Quality of Life, and Psychotropic Drug Use in Patients With Frontotemporal Dementia	N = 8	The study investigated the impact of relocating patients with frontotemporal dementia (FTD) from a traditional psychiatric ward to a group home setting with a focus on a more homelike and patient-centered approach. Before relocation, patients exhibited behavioral symptoms including disinhibition, aggression, and agitation. The study assessed cognitive function, behavioral and psychological symptoms of dementia (BPSD), quality of life (QOL), and psychotropic drug use in FTD patients over a 6-month period.	The results indicated that the group-home care approach significantly improved BPSD, as evidenced by reduced NPI and CMAI scores. Additionally, several aspects of QOL showed improvements, and the use of psychotropic drugs decreased. While further research with larger samples is needed, these findings suggest the value of a patient-centered and homelike care environment in managing behavioral disturbances in FTD patients, potentially enhancing their well-being.
Langhammer [22]	Music Therapy and Physical Activity to Ease Anxiety, Restlessness, Irritability, and Aggression in Individuals With Dementia With Signs of Frontotemporal Lobe Degeneration	N = 6 patients and 6 caregivers	In this study, an exploratory design was employed to assess the impact of an 8-week combined intervention involving physical activity, music therapy, and daily walking on individuals with severe dementia and frontal lobe symptoms in institutional care.	This study demonstrates the feasibility of implementing individualized music therapy alongside increased physical activity, indicating its efficacy in reducing behavioral symptoms associated with severe dementia.

## Data Availability

Not applicable.
